# Supplementary material for: SMOOTH protocol: A pilot randomised prospective intra-patient single-blinded observational study for examining the mechanistic basis of ablative fractional carbon dioxide laser therapy in treating hypertrophic scarring
Source: PLoS One. 2023 Sep 8;18(9):e0285230. doi: 10.1371/journal.pone.0285230 (PMC10490849; doi:10.1371/journal.pone.0285230)
Supplement: S2 File — (PDF) [file pone.0285230.s002.pdf]

SMOOTH: A prospective intra-patient Single-blinded randomised trial to examine Mechanistic basis of fractiOnal ablative carbOn dioxide laser Therapy in treating adult burns and/or trauma patients Hypertrophic scarring

Patient Information Leaflet

We would like to invite you to take part in a research study that is happening at this hospital; the SMOOTH clinical trial.

Aim of the study

Laser therapy has been used since the 1980s to treat scars, but there is limited information into how laser therapy actually works. This study aims to find out more about how the therapy works.

What is the purpose of the study?

This study aims to understand how laser treatment works on scars. We want to:

- Compare the difference between your scar after treatment with the laser to an area that was not treated with the laser
- Help improve scar appearance and the impact on your life
- Understand how this type of laser therapy works

Do I have to take part?'

No. It is up to you to decide whether or not to take part. The study is completely voluntary and by not participating this will not affect the standard of care you will receive.

We will describe the study and go through this information leaflet. If you agree to take part, we will then ask you to sign a consent form. You are free to withdraw at any time, without giving a reason.

Why have I been invited to participate?

You have been invited to participate in our study because

- You are aged 16 years and over
- You have developed a hypertrophic or thickened scar as a result of deep burns or traumatic injury that occurred more than 12 months ago
- You have a scar that has a size of at least 25cm^2 (which is less than the size of a credit or debit card)

What does it involve?

If you decide to take part, you will be asked to attend 3 laser treatments and 3 follow-up visits detailed in Table 1 (Summary of Visits). During these visits we will:

- Identify a suitable scar (5 cm x 5 cm);

- You will have laser treatment on one part of the scar and standard treatment (i.e., massage therapy, pressure garment application and/or steroid or fluorouracil injection) on the other part;
- Take some blood samples (maximum of 15mls per visit);
- Take very small skin samples from the identified scar (5 mm, equivalent to the size of phone camera lens);
- Test the thickness and the toughness of the scars;
- Ask you to complete questionnaires to assess your scars and your quality of life

Details on your involvement in the study

1. Consultation: If you agree to be part of the study, firstly we will speak to your consultant and find out which of your burn and/or trauma scars would be suitable for treatment with laser therapy in this study.

Once two areas of the scar are identified, we will use a computer tool to randomly choose which of the scar areas will be treated with the laser. The other scar will be treated with planned standard of care with your consultant. It is normal practice for your consultant to only treat one scar at a time. When the study is finished, if you want, we will also treat the other area of scarring, where laser was not used, with laser treatment.

2. Biopsies: We will need to take **seven skin biopsies** in total during four visits over the **12-month trial period.** Before your first laser therapy session, we will take 3 skin biopsies or sample (see below) . This involves taking a small 5mm (approximately the size of a phone camera lens) sample of your skin from the scars. You should not experience pain during the procedure as we will give you a local anaesthetic injection to numb the area.

Biopsies will need to be taken from different parts of the body, from scarred and non scarred skin so that we can compare normal skin with scarred skin that has and has not been treated with the laser. A sample will be taken from;

- Normal skin – an area of skin that is not scarred and not visible.
- Scarred skin that will not be treated with laser – we will call this the control area
- Scarred skin that will be treated with laser – we will call this the treatment area

These skin samples will be sent to the University of Birmingham Laboratory for analysis to see how the skin reacts to the laser treatment throughout the trial.

3. Blood Tests: We are required to take a total of **five blood samples** over the 12-month period. We will take a sample of blood (maximum of 15mls) at five different visits during the trial. This is to enable us to see if your blood has some of the same biological markers we find in your scar These samples will also be sent to the University of Birmingham Laboratory for analysis.

4. Scar Assessments: Finally, we will complete some scar assessments at four different visits during the trial. We have listed them below. You may have completed some of these before with your therapist in your outpatient appointments.

3D Photograph – this is like a normal camera but it will take a 3D image of your scar. It will help us see the dimensions of the scar and accurately and quickly measure any improvement

in the volume of the scar after laser. The photographs will be taken in such a way that you will not be identified.

Colorimeter – this device looks at the different colours in the scar. It will help show how much the redness of your scar has reduced after laser therapy.

Dermascan – this device is like an ultrasound scan performed on pregnant women. It will show us details about the different layers of skin found in a scar.

Cutometer – this device measures how flexible a scar is by applying a very gentle suction to the scar. It will show us the flexibility, elasticity and stiffness of the skin compared with scars.

Questionnaires – a series of four short questionnaires including patient reported outcome measures (PROMs) such as Brisbane Burn Scar Impact Profile (BBSIP), Patient and Observer Scar Assessment Scale (POSAS) and Participant Demographic Questionnaire. These questionnaires form part of the PROM sub-study. These PROMs will ask your opinion about your scar, the impact on your life and how it's healing. Some of these will be completed by you and some by your scar assessor. At subsequent visits we will also ask you to answer a few additional questions on whether you think your scar has changed (i.e., SMOOTH Known Groups PROM Validation Questionnaire). This will help us evaluate how well the PROMs are doing at capturing changes in your scar and its impact on you over time.

All of these tools are safe to use and will not cause any pain or discomfort. The time to complete all of the tests above is approximately 1-2 hours.

We have summarised in Table 1 (below) the visit schedules and what each visit would involve. These visits will be at the same time as when you see your consultant on your routine follow-up visits.

Table 1: Summary of Visits

	**Before your 1st Laser Treatment**	**Day of 1st Laser Treatment**	**3 weeks After 1st Treatment**	**Day of 2nd Laser Treatment**	**Day of 3rd Laser Treatment**	**6 months after 3rd Laser Treatment**
Laser Treatment		x		x	x	
Scar Assessment	x			x	x	X
Blood sample	x		x	x	x	X
Number of skin biopsies (see below)	3	0	1	0	1	2
Location of biopsies at each visit						
Healthy skin	x					
Scar treated with Laser	x		x		x	X
Scar treated with standard of care	x					X

What are the possible benefits of taking part?

We cannot guarantee the effect of the laser on your scar but there is good evidence of the benefit to scarring including the potential to:

- Improve scarring
- Reduce surgical procedures
- Improve quality of life

In addition to helping improve the appearance of your scar, you will be contributing to research that will help patients with similar scars as yours.

What about my expenses?

You will not receive any expenses or payments for being involved in the study or for attending the outpatient appointments. However, if there are attendances that are extra to your routine appointments then travel expenses will be reimbursed.

What are the possible disadvantages and risks of taking part?

The risks of this trial to your health are small. The main disadvantages of you taking part are the discomfort caused in taking blood samples and skin biopsies.

The blood sample may cause you some discomfort whilst the blood is being taken, but the procedure should only last a minute. There may be some slight bruising from where we have taken the blood but this will disappear in a few days.

The skin biopsies will be taken from your burn and/or trauma scar. You will initially feel some discomfort, which is described as a bee sting when the area is being numbed with local anaesthetic. This does not last very long, and you should not experience pain during the biopsy. The biopsies will heal quickly (usually in 2 weeks), and should not cause any extra scarring if left to heal. There is minimal risk of infection associated with a skin biopsy.

The laser treatment is safe and adverse reactions are rare. During the laser therapy, some patients can experience some pain and discomfort but this will be minimised through the application of local anaesthetic to numb the treatment area.

Scars that have not been selected to be treated with laser will be treated with standard of care for scarring. Upon completion of the trial, if you wish to have this and indeed other areas of your scar treated with laser your consultant will do so.

Who is organising and funding the research?

The University Hospitals Birmingham NHS Foundation Trust (UHBFT) is the sponsor for this study. This research project is being partly funded by UHBFT and mainly by the Scar Free Foundation, a national charity who funds research to improve the quality of life for those people living with disfigurement. We will be using information from you and/or your medical records in order to

undertake this study. UHBFT will act as the data controller for this study. This means that UHBFT is responsible for processing your information for research.

This research is being run by Professor Naiem Moiemen, a Burns and Plastics Consultant employed by the University Hospital Birmingham NHS Foundation Trust. As the Chief Investigator of the trial, Professor Naiem Moiemen is the person designated overall responsibility for the design, conduct and reporting of a study.

QEHB will keep your name, NHS number and contact details confidential and will not pass this information outside the Trust. QEHB will use this information as needed to contact you about the research study and make sure that relevant information about the study is recorded for your care and to oversee the quality of the study.

Certain individuals from the UHBFT and regulatory organisations may look at your medical and research records to check the accuracy of the research study. Your information and data will be allocated a trial specific number (pseudonymisation) for analysis . The people who analyse the data collected for the study will not be able to identify you and will not be able to find out your name, NHS number or contact details.

UHBFT will keep identifiable information about you from this study for 15 years after the study has finished/until 2034.

If you would like to find out more about why and how patient data is used in research, please visit: https://understandingpatientdata.org.uk/what-you-need-know.

Will my taking part in this study be kept confidential?

Once you have agreed to be involved in the study you will be allocated a trial specific number. We will not use any identifiable information such as your name, address or date of birth.

Any data we collect throughout the duration of the study will be stored securely at the **QEHB** and will only be viewed by the study team with the exception of authorised people who will check that the study is being carried out correctly.

Anyone who is involved in the study or anyone who monitors the conduct of study will have a duty of confidentiality to you as a research participant and we will do our best to meet this duty.

Information sent from the QEHB to the UHBFT will be securely stored and only used for this research project. The study will keep your information confidential, therefore your name will not be mentioned in any report of this research.

You can find out more how we use your information at https://www.uhb.nhs.uk/privacy-notice/research.

What will happen to my samples I give?

If you agree to be part of the trial we will collect blood and skin samples over 18 months. All of these samples will be kept securely in the University of Birmingham (UoB) Research Laboratories located at the Queen Elizabeth Hospital Birmingham, UHBFT. The UoB lab will initially run an internal rapid

testing to ensure that you are negative of COVID-19 before they proceed in processing your samples for research. If the blood results turn out to be negative, we will proceed with the study procedures. Otherwise, your samples will be discarded and you will receive a full COVID-19 swab test and we will request you to self-isolate for 14 days.

The samples you give will be pseudonymised and will only be accessed by members of the research team.

We will also ask your permission to store these samples for future research that may be carried out; if you agree to this the samples will be kept securely for a maximum of 5 years. The tissue samples would be transferred/stored in the Human Biomaterials Resource Centre (HBRC) and destroyed in accordance with the Human Tissue Act 2004.

If you do not wish to use your samples for anything other than this research study we will dispose of the samples relating to you at the end of the trial, whilst following the strict guidelines set out by the Human Tissue Act 2004.

What will happen if I don't want to carry on with the study?

You can withdraw from the study at any time, without this having any effect on your medical care.

Your data and samples that have already been collected may still be used or you can request that all your information and samples are destroyed.

In case your general health changes, such that you are no longer able to make informed decisions about your treatment, you will be withdrawn from the study. Any identifiable data or tissue already collected with consent will be retained and used in the study. Following withdrawal from the study, no further data or tissue will be collected or any other research procedures will be carried out.

We need to manage your records in specific ways for the research to be reliable. This means that we won't be able to let you change the data that we hold about you.

You may also be withdrawn from the study if:

- You become pregnant
- You have an adverse reaction to laser treatement and/or
- You are unable to complete all laser treatments as planned.

What if relevant, new information becomes available during my time in the study?

It is unlikely that this will happen during the study but if this does happen your research doctor will tell you and discuss with you whether you should continue in the study.

What will happen to the results of the research study?

The results will be published in medical journals and presented at medical conferences. All the information we present will continue to be anonymous.

Once all the information and data we have collected has been analysed, the results will be reported in a patient newsletter and disseminated to all participants of the study. Given this research is delivered by your local research team, **SRMRC Trauma and Burns Research** will have your contact details (i.e., name and address) to send you this information. If you wish to have more information about the results of the study, please inform a member of the Research Team and we can ensure that we keep you updated.

Who has reviewed the study?

All research in the NHS is looked at by an independent group of people, called a Research Ethics Committee, to protect your interests and make sure the research is safe and ethical.

This study has been reviewed and given a favourable opinion by the North of Scotland Research Ethics Committee (1).

How can I get more information on this research?

If you would like an independent opinion about the study, you may contact **Mr. Azzam Farroha** on 0121 371 4873. Mr. Farroha is a medical professional who has knowledge of the trial and treatment of scars and has no conflict of interest with the study.

If you want to speak or learn about patient experience in taking part in research, you may see the testimonies from patients at (https://www.nihr.ac.uk/about-us/our-contribution-to-research/how-we-involve-patients-carers-and-the-public.htm).

If you require any further information please don't hesitate to contact the Research Team on 0121 371 4242.

If you would prefer to speak to an independent person regarding the trial please contact the Patients Advice and Liaison Service (PALS) on 0121 371 3280.

What if something goes wrong?

In the event that something does go wrong and you are harmed during the research and this is due to someone's negligence, then you may have grounds for a legal action for compensation against University Hospitals Birmingham NHS Foundation Trust (UHBFT), but you may have to pay your legal costs.

Emergencies – If you become unwell following your discharge home from hospital you should contact your GP or attend your nearest Emergency Department. You should inform your doctor that you are in a research study and they can contact us on 0121 371 4242.

Complaints - If you have a concern about any aspect of the study, you should ask to speak to a member of the research team, who will do their best to answer your questions and concerns, on 0121 371 4242.

There are further contact details for the Research Team at the end of this Information Leaflet.

If you remain unhappy and wish to make a formal complaint you can do this by contacting the Patient Advice and Liaison Service (PALS) on 0121 371 3280.

If you wish to raise a complaint on how we handled your personal data, you can contact our Data Protection Officer who will investigate the matter. Our Data Protection Officer is Berit Reglar and you can contact them at Berit.Reglar@uhb.nhs.uk.

If you are not satisfied with their response or believe they are processing your personal data in a way that is not lawful you can complain to the Information Commissioner's Office (ICO) following this link: https://ico.org.uk/make-a-complaint/ or call their helpline on **0303 123 1113**.

Independent Contact Person

Mr Azzam Farroha
Consultant Burn and Plastic Surgeon
Queen Elizabeth Hospital Birmingham (QEHB)
Mindelsohn Way, Birmingham B15 2TH
Telephone No. 0121 371 4873

Contact Details for Research Team

Chief Investigator

Professor Naiem Moiemen
Consultant Burns and Plastics Surgeon
Burns Centre
University Hospitals Birmingham NHS Foundation Trust
Edgbaston
Birmingham
B15 2WB
Tel: 0121 371 4242/ 07747 101 720
Email: naiem.moiemen@uhb.nhs.uk

SRMRC Research Nurses
Tel. 0121 371 4242/ 07747 101 720
