# Supplementary material for: SMOOTH protocol: A pilot randomised prospective intra-patient single-blinded observational study for examining the mechanistic basis of ablative fractional carbon dioxide laser therapy in treating hypertrophic scarring
Source: PLoS One. 2023 Sep 8;18(9):e0285230. doi: 10.1371/journal.pone.0285230 (PMC10490849; doi:10.1371/journal.pone.0285230)
Supplement: S3 File — (PDF) [file pone.0285230.s003.pdf]

SMOOTH: A prospective intra-patient Single-blinded randomised trial to examine Mechanistic basis of fractiOnal ablative carbOn dioxide laser Therapy in treating adult burns and/or trauma patients Hypertrophic scarring

Patient Consent Form

Patient ID: ____________________

Please initial the box

1. I confirm that I have read and understand the Patient Information Leaflet (date:______/version:_____) for the above study. I have had the opportunity to consider the information, ask questions and have had these answered satisfactorily. ☐

2. I understand that my participation is voluntary and that I am free to withdraw at any time without giving any reason, without my medical care or legal rights being affected. ☐

3. I understand that relevant sections of my medical notes and data collected during the study may be looked at by individuals from regulatory authorities or from the NHS Trust. This is only where it is relevant to my taking part in this research. I give permission for these individuals to have access to my records. ☐

4. I understand that I will be required to give blood samples and skin samples during the study. I understand that these samples will be stored in a pseudonymous way at the University of Birmingham and once they are no longer required they will be destroyed in accordance with the Human Tissue Act (2004). ☐

5. I understand that my blood samples will initially be tested for COVID-19 and if the results turn out to be positive, my blood samples will be destroyed in accordance with the Human Tissue Act (2004). ☐

6. I consent to a photograph of the scar being captured. I understand that I will not be identified by this photo. ☐

7. I understand that I will be required to complete some questionnaires during the study about my scar, the impact of the scarring on my wellbeing and how I think my scar is healing. I understand I will be required to attend follow-up appointments to complete these questionnaires. ☐

Optional

Please initial the box

8. I agree that the tissue samples collected will also be used for future research. Yes ☐ No ☐

When completed, please make 2 copies: 1) for participant and 2) for the Medical notes; Original to be kept in Investigator Site File.

9. I understand that the questionnaires will also be used for the PROMS sub-study and the information collected will be transferred pseudonymously to the University of Birmingham for analysis. ☐

10. I understand that the information collected about me will be used to support other research in the future, and may be shared anonymously with other researchers. ☐

11. I agree to take part in the above study. ☐

________________________	____________________	________________________
Name of Patient	Date	Signature
________________________	____________________	________________________
Name of Person taking consent	Date	Signature

When completed, please make 2 copies: 1) for participant and 2) for the Medical notes; Original to be kept in Investigator Site File.
