# Supplementary material for: SMOOTH protocol: A pilot randomised prospective intra-patient single-blinded observational study for examining the mechanistic basis of ablative fractional carbon dioxide laser therapy in treating hypertrophic scarring
Source: PLoS One. 2023 Sep 8;18(9):e0285230. doi: 10.1371/journal.pone.0285230 (PMC10490849; doi:10.1371/journal.pone.0285230)
Supplement: S4 File — (DOCX) [file pone.0285230.s004.docx]

**SMOOTH**

A prospective intra-patient **S**ingle-blinded randomised trial to examine the **M**echanistic basis of fracti**O**nal ablative carb**O**n dioxide laser **T**herapy in treating adult burns and/or trauma patients with **H**ypertrophic scarring

**Study Reference Code:** SMOOTH

**IRAS Project ID:** 262030

**RRK Number:** 6716

**REC No.:** 19/NS/0125

**CPMS ID. :** 42618

**Version Number:** 7

**Date:**  22^nd^ September 2022

**Sponsor Name & Address:** University Hospital Birmingham Foundation Trust.

Mindelsohn Way,

Edgbaston

Birmingham

B15 2WB

# VERSION CONTROL LOG

*The table below provides an overview of the summary of changes from previous version of this protocol. Where no previous version exists there will be no summary of changes on record.*

| **Version number** | **Date** | **Summary of changes** |
| --- | --- | --- |
| 1 |  | N/A |
| 2 | 29/07/2019 | Removal of punch biopsy on second laser treatment |
| 3 | 13/11/2019 | Contact Details: Additional co-PI for the Morriston Hospital and identifying the independent contact person as previously required by REC  2.2.1 Primary endpoints - deletion of some other biomarkers that will not be identified such as SASP, sequencing apoptotic markers, epigenomics  2.2.2 Secondary endpoint - deletion of 2D since the camera to be used is 3D  3.1 Diagrammatic Overview of Study Design (Figure 1)- The 12 month follow-up commences after the 3rd laser treatment and not after the first laser treatment. Relevant sections of the protocol are amended such as section 7.5.6 on 12-month follow-up visit and Appendix A (Study Schedule).  4.3 Inclusion criteria - adding the word symptomatic on item 2  4.4.1 Laser Exclusion criteria - stating the generic name on item 6 and removal of Type 1 Diabetes Mellitus on item 7 since both Type 1 and Type 2 diabetic patients can be treated.  5.1 Identifying participants - added the specific clinic at Morriston hospital where patients will be seen  5.2.1 changing "Additional psychometric group" sub-title to "PROMS Sub-Study" and clarifying how PROMS Sub-study will be delivered alongside the SMOOTH study.  6.2.1 Control Area - clarifying that patients may opt to undergo laser treatment after the trial is completed  6.2.2 Treatment Area - Specifying the laser settings  7.1.1 Risk - Correcting the period when the expected risk will be resolved  7.3.3.1 Providing additional details on how tissue samples will be handled and stored  7.3.5.1 DSM II is replaced by DSM III as this is the latest available device- Providing additional details about the devices and removing the additional requirement to blanch scars before measurement; blanching scar is not necessary for this study  7.3.5.2 Additional clarification on the use of the device and to include density measurement.  7.4.1 Laboratory assessment - added statement on how biopsies will be handled and stored  7.4.1.1 Minor correction (typo error) on the location where biopsies will be taken.  7.4.1.2 Analysis of blood - changing the volume of serum and plasma to be collected but the total volume of 10mls remain. Clarifying how plasma will be processed.  8.3.1 Added a paragraph on the sample size for the PROMS Sub-study and justifying the significance for the required sample size  10. Amending the funding source details. Insurance was amended as per recommendation of UHB R&D Head of Governance.  12. Archive Plan was revised in accordance with the UHBFT Research Archiving Policy. |
| 4 | 14/02/2020 | 4.4 Exclusion criteria: reducing the timepoint from concurrent invasive scar treatments from 6 months to 3 months as long as it is not on the same area identified for the study.  6.2. Treatment Area: Increased upper limit for laser energy setting to 20mJ; adding statement on carrying out laser treatment on other sites in addition to the randomised scar  7.4.1.2 Analysis of Blood: Increase blood sample volume from 10 mls to 15mls for Morriston Hospital in Swansea due to processing capability of their blood analyser. |
| 4.1 | 15/10/2020 | 7.4.1 The UoB laboratory will initially process the bloods and run a rapid testing for COVID-19 as a safety measure before they proceed in processing bloods for research. UoB lab is currently processing confirmed COVID-ve bloods. If the blood results turn out COVID+ve, the blood samples will be discarded in accordance with the Trust policy in disposing biological samples.  APPENDIX A. Footnote 8 - additional 1 ml blood green top lithium heparin for COVID-19 testing. |
| 4.2 | 23/06/2021 | 6.2 Laser settings; no additional laser treatment will be done on other areas on the duration of the study. (added in version 6.0, classed as substantial amendment) |
| 5.0 | 06/07/2021 | Moving forward the final follow-up time point from 12 months after last laser treatment to 6 months; amended the study outline, study schedule and other relevant sections of the study accordingly. |
| 6.0 | 26/07/2021 | 6.2.2 Amended the laser settings to make it less painful for the patient; no additional laser treatment will be done on other areas outside the randomised scars throughout the study period.  Updated the key study contacts list and details |
| 6.1 | 15/07/2022 | Correction oftypographical errors and addition of current research staff |

# PROTOCOL SIGNATURE SHEET

**This protocol has been approved by:**

| **Name & Address:**  <*print – delete this text on completion>* | **Role:**  **Sponsor Representative** |
| --- | --- |
| **Signature:** | **Date:** |

| **Name & Address**  <*print name – delete this text on completion>* | **Role:** |
| --- | --- |
| **Signature:** | **Date:** |

**Principal Investigator Declaration:**

I have read and understood the requirements and conditions of the study protocol. I am aware of my responsibilities as an Investigator under the guidelines of the Internal Conference on Harmonisation Good Clinical Practice (ICH GCP) standards, the Declaration of Helsinki, local regulations (as applicable) and the study protocol. I agree to conduct the study according to these guidelines and to appropriately direct and assist the study team assigned to me who will be involved in the study.

I agree to use the study material, including medication, only as specified in the protocol.

I understand that changes to the protocol must be made in the form of an amendment that must be approved by the Ethics Committee and Regulatory Authorities prior to its implementation.

I understand that non-compliance with the study protocol may lead to early termination of the study.

| **Local Investigator’s Name & Address:**  <*print name – delete this text on completion>* | |
| --- | --- |
| **Signature:** | **Date:** |

Return the original wet signature* page to the sponsor and retain a copy* with the study protocol within the Investigator’s Site file.

* *Reference to wet signature means signature in ink, copy can be a scanned copy or photocopy of the signature page.*

# KEY STUDY CONTACTS

**Chief Investigator:**

**Professor Naiem Moiemen**

Consultant Burns and Plastic Surgeon

Director, Healing Foundation Centre for Burns Research, Birmingham, UK

Queen Elizabeth Hospital Birmingham (QEHB)

Mindelsohn Way, Birmingham B15 2TH

Telephone: 0121 371 3747

[**naiem.moiemen@uhb.nhs.uk**](mailto:naiem.moiemen@uhb.nhs.uk)

**Principal Investigators:**

**Professor Janet Lord**

Professor of Immune Cell Biology

University of Birmingham

0121 3713234

[**j.m.lord@bham.ac.uk**](mailto:j.m.lord@bham.ac.uk)

**Mr Max Murison**

Consultant Laser and Plastic Surgery

Welsh Centre for Burns and Plastic Surgery

Morriston Hospital

Swansea

SA6 6NL

[Max.Murison@wales.nhs.uk](mailto:Max.Murison@wales.nhs.uk)

**Mr Jeremy Yarrow**

Consultant Laser and Plastic Surgery

Welsh Centre for Burns and Plastic Surgery

Morriston Hospital

Swansea

SA6 6NL

**Miss Yvonne Wilson**

Consultant Plastic Surgeon

Birmingham Children Hospital

Steelhouse Lane, Birmingham B4 6NH

[**yvonne.wilson26@nhs.net**](mailto:yvonne.wilson26@nhs.net)

**Mr Azzam Farroha (Independent Contact Person)**

Consultant Burn and Plastic Surgeon

Queen Elizabeth Hospital Birmingham (QEHB)

Mindelsohn Way, Birmingham B15 2TH

**Dr Anita Slade**

PhD, MPhil, DipCOT

ITM Research Fellow

Centre for Patient Reported Outcomes Research

Institute of Applied Health Research

College of Medical and Dental Sciences

University of Birmingham

Edgbaston

B15 2TT

**Dr Jon Bishop**

Senior statistician

Birmingham Clinical Trials Unit

Institute of Applied Health Research

Public Health Building

University of Birmingham

Edgbaston

B15 2TT

**Mrs. Amy Bamford**

Lead Nurse - Research

NIHR SRMRC

ITM Building

Queen Elizabeth Hospital Birmingham (QEHB)

Mindelsohn Way, Birmingham B15 2TH

[amy.bamford@uhb.nhs.uk](mailto:amy.bamford@uhb.nhs.uk)

**Researchers & Co-Investigators:**

**Mr Ezekwe Amirize**

Burns Clinical Research Fellow

University Hospitals Birmingham NHS Foundation Trust

[Ezekwe.Amirize@uhb.nhs.uk](mailto:Ezekwe.Amirize@uhb.nhs.uk)

0121 371 4242

**Mr Abdulrazak Abdulsalam**

Burns Clinical Research Fellow

University Hospitals Birmingham NHS Foundation Trust

Abdulrazak.Abdulsalam@uhb.nhs.uk

0121 371 4242

**Amberley Prince**

Senior Occupational Therapist

Queen Elizabeth Hospital Birmingham

Mindelsohn Way, Birmingham B15 2TH

Amberley.prince@uhb.nhs.uk

**Study Co-ordinators**

Laura Mee

Clinical Trials Coordinator

REACT Team (formerly NIHR-SRMRC)

University Hospitals Birmingham NHS Foundation Trust

[Laura.Mee@uhb.nhs.uk](mailto:Laura.Mee@uhb.nhs.uk)

0121 371 4242

**Patient & Public Involvement**

**Dr Laura Nice**

Patient & Public Involvement Lead

NIHR SRMRC - Trauma Research

Centre for Conflict Wound Research

#

# TABLE OF CONTENTS

VERSION CONTROL LOG 2

PROTOCOL SIGNATURE SHEET 4

Contact Details: 5

TABLE OF CONTENTS 7

LIST OF ABBREVIATIONS & DEFINITIONS 8

1. BACKGROUND INFORMATION 9

2. STUDY OBJECTIVES 10

2.1. Objectives 10

2.1.1 Primary Objectives 10

2.1.2 Secondary Objectives 10

2.2 Study Endpoints 10

2.2.1 Primary Endpoints 10

2.2.2 Secondary Endpoints 11

3. STUDY DESIGN 11

3.1 Methodology 11

4. STUDY POPULATION 143

4.1 Number of Participants 153

4.2 Expected Duration of Study 153

4.3 Inclusion Criteria 15

4.4 General Exclusion Criteria 15

4.4.1 Laser Treatment Exclusion Criteria 13

5. PARTICIPANT SELECTION AND ENROLMENT 164

5.1. Identifying Participants 16

5.2. Consenting Participants 16

5.2.1 Additional psychometric group 14

5.3. Screening for Eligibility 175

5.4. Randomisation 15

5.4.1 Randomisation Procedures 15

5.4.2 Treatment Allocation 15

5.4.3 Blinding 15

5.5. Removal of Patients from Therapy or Assessment 175

6. STUDY TREATMENTS 186

6.1. Treatment Schedule/Plan 186

6.2 Description of the treatment 18

6.2.1 Control Area (Standard of Care) 18

6.2.2 Treatment Area 186

6.3 Concomitant therapy 196

7. STUDY ASSESSMENTS 197

7.1 Safety Assessments 17

7.1.1 Risk 17

7.2 Safety Reporting 17

7.2.1 Serious Adverse Events 17

7.2.2 Adverse Event Recording and Reporting 17

7.2.3 Pregnancy 18

7.3 Study Assessments 18

7.3.1 Parameters 18

7.3.2 Blood Sampling 18

7.3.3 Biopsies and Samples 18

7.3.3.1 Punch biopsy Procedure 19

7.3.4 Subjective Scar Assessment 19

7.3.4.1 Vancouver Scar Scale 19

7.3.4.2 Patient and Observer Scar Assessment Scale 19

7.3.4.3 Brisbane Burn Scar Impact Profile (BBSIP) 20

7.3.4.4 EQ-5D 20

7.3.5 Objective Scar Assessment 20

7.3.5.1 DSM II Colorimeter 20

7.3.5.2 Dermascan C USB 20

7.3.5.3 Cutometer 21

7.3.5.4 Vectra H1 3D Camera 21

7.4 Clinical and Laboratory Assessments 21

7.4.1 Laboratory Assessment 21

7.4.1.1 Analysis of Tissue 22

7.4.1.2 Analysis of Blood 22

7.4.2 Storage and disposal of samples 23

7.5 Schedule of Assessments and Data Collection 23

7.5.1 Baseline Data Collection (Recruitment/pre-assessment) 23

7.5.2 First laser intervention 24

7.5.3 Post 1^st^ laser intervention 24

7.5.4 Second laser intervention 24

7.5.5 Third laser intervention 24

7.5.6 Follow -up Visit 24

8. DATA MANAGEMENT & STATISTICS 275

8.1 Determination of Sample Size 25

8.1.1 Laser Treatment and Standard of Care 25

8.2 Statistical Analysis 275

8.3 Psychometric Data Analysis 286

8.3.1 The Rasch Model 26

8.3.1.1 Rasch Analysis 26

8.3.1.2 Scoring Catergories 26

8.3.1.3 Targeting 27

8.3.1.4 Person Specification Index 27

8.3.1.5 Tests of fit 27

8.3.1.6 Differential Item Functioning 27

8.3.1.7 Item Independence 27

8.3.1.8 Logit transformation 27

8.3.1.8 Modify instrument 28

8.4 Data Monitoring & Interim Analysis 28

8.5 Direct Access to Source Data/Documentation 28

8.6 Data Handling and Record Keeping 28

8.7 Quality Control and Quality Assurance 31

9. Ethics 31

10. Finance and Insurance 31

11. Publication Policy 31

12. Archive Plan 31

**REFERENCES**  **32**

Appendix A. Study Schedule 34

# LIST OF ABBREVIATIONS & DEFINITIONS

| **Abbreviation** | **Definition** |
| --- | --- |
| AE | Adverse Event |
| BBSIP | Brisbane Burn Scar Impact Profile |
| CASEVAC | The Casualty Evacuation Club |
| CO_2_ | Carbon Dioxide |
| CTT | Classical Test Theory |
| eCRF | Electronic Case Report Form |
| GCP | Good Clinical Practice |
| KTP | Key Performance Indicator |
| PIL | Patient Information Leaflet |
| POSAS | Patient and Observer Scar Assessment Scale |
| PROM | Patient Reported Outcome Measures |
| QEHB | Queen Elizabeth Hospital Birmingham |
| SAE | Serious Adverse Event |
| SOP | Standard Operating Procedure |
| TBSA | Total Body Surface Area |
| UHB | University Hospital Birmingham |
| UoB | University of Birmingham |
| VSS | Vancouver Scar Score |
| 3D | Three Dimensional |

# BACKGROUND INFORMATION

Burn injury is the fourth most common type of trauma after road traffic accidents, falls and interpersonal intentional injury. In 2004 it was estimated that worldwide 11 million people suffered fire-related burn injury with 265,000 deaths annually. Burns are also one of the leading causes of disability-adjusted life-years lost. Crucially, up to 90% of the patients who survive a burn injury suffer significant scarring, with hypertrophic scars predominating.

Burn care during the last 30 years has seen a step change in survival and this increased survival has been paralleled by improved acute care and durable wound cover resulting in less deformity and scarring. However, there remains an urgent need for improvements in post-burn scar assessment, management and the treatment of historic scars. Gangemi *et al* in a comprehensive review of 703 burn survivors’ records identified the key risk factors for post-burn hypertrophic scarring (1).

These factors included being a young person, female, having dark skin, sustaining a severe burn, number of surgical procedures performed to achieve wound cover, site on the body where the burn occurred and time to wound healing. How these factors influence the treatment of established scars remains poorly understood.

Post burn hypertrophic scarring is typically treated non-invasively with the use of moisturiser, massage, pressure garments, silicone or these modalities in various combinations. A survey of 19 paediatric burn services in the UK showed that 18 services routinely use pressure garments for prevention of hypertrophic scarring following burn injury. More recently injection into the scar of steroids or anti-neoplastic drugs such as Fluorouracil, Bleomycin and Interferon have come into use. Other commonly used drugs include verapamil and botulinum toxin type-A, which have been reported to be beneficial if injected either alone or in combination with steroids.

Laser therapy for treating hypertrophic scarring is a relatively new concept started in the 1980s, being used initially to treat port-wine stains and remove decorative tattooing. Although its use is becoming more widespread for the reduction of established scars its efficacy and mechanism of action remain to be established.

Three main methodological variants of laser therapy have been developed over the years to treat specific aspects of established scars: Pulsed dye lasers, Q-switched Nd:YAG lasers and Fractional lasers. Pulsed dye laser therapy was used to reduce scar vascularity by inducing disruption of the targeted capillaries. This method was also reported to reduce itch. Nd:YAG lasers emit light in the infra-red range, typically with a wavelength of 1064nm and have deeper tissue penetration. They have a range of applications including facial hair and nevus removal.

Fractionated CO2 laser therapy was introduced by Manstein et al in 2004 and essentially bridges the gap between the ablative and non-ablative laser techniques (2). Ablative laser treatments work mainly on the epidermis and non-ablative treatments work solely on dermal collagen, fractional laser treatment works at both the epidermal and dermal layers of the skin making it suitable for treating several aspects of hypertrophic scarring.

Twelve RCTs of laser therapy for the treatment of hypertrophic scars, involving 592 patients, were considered recently in a systematic review (3). Although 11 out of 12 of these trials reported a positive effect of the therapy the review concluded there was insufficient evidence of the clinical effectiveness of laser therapy. This was largely due to variations in the laser therapy used, the scar assessment methods selected and poor study design with a high level of bias. Currently there are 3 RCTs open in Canada and one in the US, this promising new therapy thus remains to have its clinical efficacy in scar management proven.

We suggest that improved understanding of the mechanisms that mediate scar reduction with laser treatment, combined with standardised assessment of scarring and rigorous RCT design, will enable better design of laser treatment regimens and thus benefit those living with hypertrophic scarring.

# STUDY OBJECTIVES

## Objectives

- - 1. **Primary Objectives**

To assess the kinetics of the response to fractionated CO_2_ laser therapy in hypertrophic scars, in particular to test the hypothesis that a positive effect on the scar (assessed through objective and subjective measures) is associated with reduced number of myofibroblasts and the increased presence of senescent skin cells.

- - 1. **Secondary Objectives**

To determine associations between the various cellular and molecular biomarkers and reduction in scarring post laser therapy.

To evaluate the extent to which the use of Brisbane Burn Scar Impact Profile (BBSIP) is a sound psychometric measure of improvements in scarring and the impact on participant’s quality of life as a result of laser treatment.

## 2.2 Study Endpoints

**2.2.1 Primary endpoints**

The primary endpoint is to assess the effect of the ablative carbon dioxide laser therapy via histological assessment of the hypertrophic scarring and in particular using novel methodology to detect the number of senescent cells and the subpopulation of fibroblast 6 months after the third laser treatment.

Scars will be assessed histologically (5mm punch biopsy) to assess dermal and epidermal thickness, collagen structure and orientation, elastin density and structure. Immunostaining will examine possible scar biomarker, proportion of senescent cells and the proportion and sub-population of fibroblasts following treatment.

To identify novel markers associated with scarring and tissue regenerative capabilities such as markers of cellular senescence and DNA damage, for example p16 and ɣH2Ax and other biomarkers including: micro RNAs, TGF-beta, decorin, VAP-1, MMPs, PDGF, adiponectin, etc.

To identify expressions and markers both in blood and tissue samples that influence scar formation, wound healing and variables of scar behaviour in response to CO_2_ laser therapy. This will include; pro and anti-inflammatory cytokines, scar biomarkers and markers of cellular senescence, for example mmp.

**2.2.2 Secondary endpoints**

The secondary endpoints include: subjective (BBSIP, POSAS and mVSS) scores and objective assessment of the areas of interest on Months 3, 6 and 12 after first laser treatment. Objective assessment includes; 3D (Vectra H1) photography, scar thickness and density using Ultrasound (Derma scan cortex Denmark), pliability (Cutometer) and scar colour and pigmentation using DSM Colorimeter .

# STUDY DESIGN

**3.1** **Methodology**

This is a multicentre intra-patient randomised controlled longitudinal observational cohort study, with parallel assignment, single blinded clinical trial to test the effect of ablative fractionated CO_2_ laser on hypertrophic scarring. Two anatomically comparable and independent scars would be selected per subject and randomly allocated to receive either fractioned CO_2_ laser therapy or standard care. An independent assessor will be blinded to the intervention and the control scars sites.

The study will collect quantitative data through tissue and blood samples to assess the cellular and molecular biomarkers response to laser therapy and qualitative data using a series of patient reported outcome measures (PROMs) and scar assessments. Scars will be subjectively assessed using the standard of care scar assessments; Modified Vancouver Scar Scale (mVSS), Patient and Observer Scar Assessment Scale (POSAS)*,* Brisbane Burn Scar Impact Profile (BBSIP). Scars will also be objectively assessed using 3D Vectra Photography for scar site identification, high resolution ultrasound for thickness, Cutometer^®^ for elasticity and colour using DSMII Colorimeter® camera.

As part of the evaluation of the impact of laser treatment on patients’ quality of life a PROMS validation study will be carried out to evaluate if the BBSIP is a useful tool for evaluating the impact of laser therapy on scar tissue and quality of life.

PROMs describe how the patient is functioning or feeling without input from clinical staff (17) providing a unique perspective of patients’ lived experience of the disease as not all symptoms or impacts are obvious to clinicians (18). In order for PROMs to be effective in clinical trials and practice, they have to capture information on domains that matter to the patient (17).

A previous study carried out in Birmingham (13) identified that there were a number of important domains for patients with burn injuries and these were applicable when evaluating relevant interventions. These include: scarring, movement and function, scar sensation, psychological distress, body image and confidence, engagement in activities, treatment burden and impact on relationships.

The study also identified that only two measures had been developed with patient input, in line with FDA recommendations, and only one that was scar specific. The Brisbane Burn Scar Impact Profile (BBSIP) will be used in this study to evaluate its psychometric properties in patients receiving laser therapy. The tool will be validated in this population using Classical Test Theory (CTT) and Rasch Analysis, alongside a range of questions to establish the ability of the PROM to differentiate between groups e.g. improved scar tissue, no change or worse scarring and clinical data.

BBSIP data will be collected in clinics as illustrated in the diagram below (Figure 1), and patients will be given the option to complete a paper and pencil version of the measure or an online version using smart survey. Participants will also be asked to sign a consent to contact form to enable a member of the CPROR research team to analyse their data and have access to information from the demographic questionnaire for use in the psychometric analysis.

Participants to the study will be recruited at the outpatient clinics from the Queen Elizabeth Hospital Birmingham and the Morriston Hospital, Swansea.

Patients deemed suitable for the research trial and who meet the inclusion and exclusion criteria will be assessed for capacity before proceeding with the informed consent process.

**Diagrammatic Overview of Study Design (Figure 1)**

1. **STUDY POPULATION**

## Number of Participants

A total of sixty (60) participants will be recruited to the study over the period of 2 years. Civilian patients will be recruited from the recently established Advanced Scar Management clinic at University Hospital Birmingham and The Welsh Centre for Burns and Plastic Surgery, Morriston Hospital, Swansea. Veterans will be recruited in collaboration with the CASEVAC Club volunteers.

- 1. **Expected Duration of Study**

We anticipate that the recruitment and follow up of 60 patients across both sites will be 2 years or after the 6-month follow-up of the last patient.

- 1. **Inclusion Criteria**

1. Adult patients aged ≥ 16years
2. Patient with symptomatic hypertrophic scarring as a result of deep dermal or full thickness burns/trauma.
3. Trauma or Burn sustained more than 12 months prior to recruitment.
4. Treatment area to be ≥25cm^2^ confluent scarring with a comparable control scar on limb or trunk

## 4.4 General Exclusion Criteria

1. Patients under 16 years of age
2. Previous laser therapy treatment to the study site
3. The use of recent (within 3 months) or concurrent invasive scar treatments, including intra-lesional pharmaceuticals, micro needling or other laser modalities (e.g. Pulse-dye.) on the study site.
4. Known allergy or contraindication to EMLA™ 5% Cream (Lidocaine 2.5% and Prilocaine 2.5%), Dermol 500^TM^ (Benzalkonium Chloride 0.1%; Chlorhexidine Dihydrochloride 0.1%; Liquid Paraffin 2.5%; Isopropyl Myristate 2.5%) or 50:50 ointment (White Soft Paraffin Liquid Paraffin %w/w 50 50.)
5. Patients with Fitzpatrick skin type of 5-6 due to nature of the skin

(The concurrent use of pressure garments, emollient application and scar massage will not exclude enrolment to this study.)

**4.4.1** **Laser Treatment Exclusion Criteria**

1. The presence of acute infection at the proposed treatment site
2. Pregnancy or lactation
3. Patients with poorly controlled Diabetes mellitus HbA1C >9% or 75mmol/mol within last 3 months)
4. Patients experiencing acute exacerbation of Chronic skin diseases e.g. psoriasis or eczema
5. Immunosuppression (HIV, drugs with immunosuppressive effect)
6. Use of Isotretinoin (Roaccutane) at any time within the last 6 months
7. Autoimmune disorders in active stage (for example: 1. Localised: Addison’s, Grave’s and Crohn’s Disease, 2. Systemic:Rheumatoid Arthritis, Multiple Sclerosis, Lupus and Scleroderma).
8. Known history of keloid scarring
9. **PARTICIPANT SELECTION AND ENROLMENT**

## Identifying Participants

Patients will be identified through the Advanced Scar Management clinic, burns and scar therapist outpatient clinics at QEHB and consultants/therapy led clinics at Morriston Hospital, Swansea. Patients may be approached in person by a member of the research team or via telephone during which a brief explanation of the trial will be given.

If the patient agreed, a Patient Information Leaflet (PIL) will be sent by mail or given to the patient.

## Consenting Participants

Written informed consent will be obtained from all subjects before the start of the study related procedure. The research team will assess patient’s eligibility for each treatment intervention, i.e., first, second and third laser treatment, and re-confirm patient’s consent if they agree to continue with the treatment in each time point.

**5.2.1** **PROMS Sub-Study**

Patients attending routine clinical appointments will be asked if they are willing to complete copies of the BBSIP and POSAS plus a consent form and demographic questionnaire to enable psychometric validation of the PROMs. Inclusion criteria will be any patients with sufficient understanding of written English to complete a PROM. Exclusion criteria will be patients with insufficient written English to complete PROMs. Data will be collected over a two-year period in line with the laser study data collection time points. Patients recruited for the SMOOTH study will not be taking part in the PROMS sub-study since the same data will be collected in the SMOOTH study. SMOOTH patients will only be asked to sign one consent form to take part in the study and will complete the PROMs as part of the SMOOTH study.

The PROMS PIS and validation consent form will be used to recruit additional patients who are not part of the SMOOTH study to test the psychometric validity of the BBSIP and POSAS. As only 60 patients will be recruited to the SMOOTH study and this is not sufficient to test the psychometric validity of the PROMs being used. The sub-study will be carried out in routine clinics alongside the SMOOTH study. Data will be collected at two time points and patients will be asked to complete the known groups PROM validation questionnaire at the second time point. This allows the investigators to see if changes captured by the PROMs reflect participants perceptions of change in their scar tissue.

The investigators have both ethical and legal responsibility to ensure that each subject being considered for inclusion in this study is given a full explanation of the protocol and has demonstrated their understanding. This shall be documented on written informed consent forms

An ethically approved written informed consent will be signed and dated by: the subject (or the subject’s legally authorized representative), the individual obtaining consent, and by any other parties required by the ethics committee. This will be completed after the appropriate and essential information has been provided to the participant) and has been fully explained by the investigator (or designee), and it is felt that the participant (or the participants’s legally authorized representative) understands the implications of participating in the study.

The subject (or the subject’s legally authorized representative) shall be given a copy of the signed informed consent form, the original copy shall be kept in the investigator site file at the study centre. A second copy will be filed in the subject’s medical record.

## Screening for Eligibility

Subjects who are deemed suitable for the trial would be identified at Outpatients burn and/or trauma clinic at Queen Elizabeth Hospital Birmingham, Morriston Hospital in Swansea and among the CASEVAC Club members. The research team will approach the participants for screening to ensure that they meet the inclusion and exclusion criteria.

## Randomisation

**5.4.1 Randomisation Procedures**

Each patient will have their own control scar in an anatomically comparable site either on the trunk or limbs. Allocation of scar treatment will be performed using randomisation. Two comparable anatomically scarred areas > 25 cm^2^ on trunk, arms or legs will be identified on the same patient and will be described as location A and location B on the body map. The patient’s identified scar site, A and B, will be randomised on a 1:1 basis to either the standard of care or laser treatment as shown in Table 1.

**5.4.2 Treatment Allocation**

A randomisation list (Table 1) will be prepared before the start of the study and will be implemented using a computer-based randomisation system developed at UHBFT . Once randomised, the treatment allocation will be as follow:

**Table 1.** Options for assignment of study areas

| **Option** | **Treatment allocation for Location A** | **Treatment allocation for Location B** |
| --- | --- | --- |
| Option 1 | Laser Treatment | Standard of care |
| Option 2 | Standard of Care | Laser |

**5.4.3** **Blinding**

Scar assessment will be performed by an independent and experienced assessor in scar management and treatment. This health care professional or scar assessor, in addition to the data analysts will be blinded to the anatomical site (treatment area) receiving laser treatment compared to the non-laser treated scar (control area).

## Removal of Patients from Therapy or Assessment

Subjects may withdraw or be withdrawn from the study at any time.

Patients may be withdrawn from the study if an incidence occurs that renders them unable to continue with the study. For example:

- Patients not able to complete all Laser Therapy Sessions as planned
- Adverse reaction to Laser Therapy
- Pregnancy

The reason for discontinuation will be collected and recorded in the electronic case report form (eCRF). Any data collected at the time of withdrawal may still be included in the data analysis, unless the participant specifically withdraws their consent. Patients will be asked to clarify this at the point of withdrawal.

1. **STUDY TREATMENTS**
   1. **Treatment Schedule/Plan**

Three laser treatment time points will occur at 3 months intervals post recruitment D1, M3 (+/- 30 days) and M6 (+/- 30 days). Scar assessment for both the treatment and the control areas will be performed and recorded prior to randomisation and before each laser treatment. The control site would be treated as per standard of care and if the study subject desire laser therapy for this area, this would be treated on the 6^th^month follow-up after study completion. All study visits are aligned with standard of care treatments and no additional follow up visits would be expected.

**6.2 Description of the treatment**

**6.2.1** **Control Area (Standard of Care)**

Patients with hypertrophic scarring usually undergo massage therapy, pressure garment application and/or steroid or fluorouracil injection as part of standard practice. At present, alternative treatments include cryotherapy, microneedling, fat transfer and Laser Therapy.

As these patients have various laser therapy to different parts of the body over a period of months or years, the control scar, if deemed of inferior quality to the laser treated area, will be treated as part of the patient routine ongoing management of their scars after the trial is completed.

**6.2.2** **Treatment Area**

Participants will undergo Ablative CO2 Laser treatment as part of standard of care. Typically, the laser treatment will be performed under local anaesthetic and will be conducted by a suitably trained medically qualified doctor, as the patients have agreed to participate in the research study, the doctor will also require knowledge of the research protocol.

The treatment will use a Lumenis® UltraPulse® CO2 laser device with the DeepFX^TM^ and/or SCAARFX^TM^ headpiece depending on scar thickness assessed by ultrasound prior to treatment. The proposed treatment area will be marked and photo documented. The treatment will include a single pass of the chosen treatment site with the following settings: SCAARFX^TM^: Energy 110-150 mJ; Density 3%; Shape: setting 2; Size: setting 10; Pulses 1; and DeepFX^TM:^ Energy 17.5-20.0 mJ; Density 5%; Shape: setting 2; Size: setting 10; Pulses 1. Repeat rate rate 0.5 -1 seconds; Frequency 250-300Hz; No active cooling will be used during the treatment.

Laser treatment during the trial will only be carried out on the randomised scar. No additional laser treatment will be done on other areas outside the randomised scars throughout the study period.

**6.3 Concomitant therapy**

Control and laser treated (randomised) scars, when healed, will be treated as per Standard of Care which would include silicone, massage and pressure therapy, when applicable.

1. **STUDY ASSESSMENTS**
   1. **Safety Assessments**

**7.1.1 Risk**

The risks associated with this study are related mainly to the potential adverse effects of CO2 laser therapy. In general, the clinical consensus regarding CO2 laser treatment of hypertrophic scars is that this therapy is well tolerated, and that adverse events related to treatment are rare. Nevertheless, we intend to rigorously and prospectively monitor, record and report adverse events to protect subject safety and evaluate prospectively the aforementioned current clinical consensus

Furthermore, all subjects may experience pain at the treatment site which is is expected to resolve within 3 days. Weeping and later crusting, itch, as well as treatment-related transient skin color changes are all expected to resolve up to 6 months after each treatment.

Specific treatment-related events that each site will be instructed to look for include:

- cellulitis
- superficial thrombophlebitis
- epidermolysis
- bullous conversion of treatment site
- herpetic vesicles or shingles lesions
- hypopigmentation
- hyperpigmentation
- severe or prolonged pain
- severe or prolonged itch

Severe events, such as necrotizing soft tissue infection, as well as full-thickness dermal injury (which could require skin grafting) are anticipated to be rare.

**7.2 Safety Reporting**

**7.2.1 Serious Adverse Events**

Clinical, research team and the trial investigators will closely monitor safety of all trial subjects throughout the duration of the study. In the event that any adverse events occur they will be recorded and reported immediately according to the Standard Operating Procedures (SOP) for Research Ethics Committee (REC) version 7.4. This SOP provides the definition of a SAE. Any possible serious events that could possibly be study related will be reviewed and reported electronically within 24 hours by the study PI to the study sponsor (R&D Department, UHBFT).

**7.2.2 Adverse Event Recording and Reporting**

As this is an observational cohort study examining the effect of laser treatment which is administered as part of standard of care, pursuant to SOP for REC version 7.4, only serious adverse events (SAEs) where in the opinion of the Chief Investigator (CI) are related to the study (i.e., resulted from administration of any research procedures such a tissue biopsies or blood sampling) and unexpected (i.e., not listed in the protocol as an expected occurrence) should be reported to REC. Report of related and unexpected SAEs should be submitted to REC within 15 days upon CI’s knowledge of the event using the SAE form for non-CTIMPs published on the HRA website.

All AEs and SAEs will be recorded and reported on throughout the duration of the study. The Investigator will then record all relevant information in the CRF/SAE log.

Information to be collected includes:

- Type and details of event
- Event duration
- Investigator assessment of severity and causality and if the event was expected or unexpected
- Action taken
- Date of resolution
- Treatment required, investigations needed and outcome.

**7.2.3 Pregnancy**

Participants who become pregnant during the trial will no longer be eligible for laser treatment but can continue for follow up if they wish to do so. The patient will continue to receive standard of care.

Pregnancy will be screened but will not be routinely tested during this study.

**7.3 Study Assessments**

**7.3.1 Parameters**

Efficacy parameters to be assessed include; histological examination of scars following laser therapy, PROMS measured with BBSIP, subjective scar assessment (POSAS and mVSS) and objective scar assessment (scar thickness, density, pliability and colour)

**7.3.2 Blood Sampling**

Blood will be taken following local standard operating procedure (SOP) using venepuncture.

Blood samples will be analysed for pro and anti-inflammatory cytokines and scar biomarkers.

**7.3.3 Biopsies and Samples**

Tissue samples will be taken from the scar to be/has been treated with Laser therapy. The initial sample will provide a baseline histological profile of the scar tissue and subsequent samples will examine the changes in biomarkers associated with cellular senescence and scar formation including RNA molecules, senescent skin cells, TGF, decorin, VAPs, MMPs, PDGF, adiponectin, etc.

**7.3.3.1 Punch Biopsy Procedure**

The following procedure will be followed by the medical staff performing the punch biopsy:

- The intervention area will be injected with 1% Lignocaine (local anaesthetic) with 1:200,000 adrenaline before taking the scar biopsies. To supplement the topical anaesthetic amethocaine (Ametop) or EMLA cream 5% used before the laser therapy. The addition of local to topical anaesthesia will prevent the sharp pain that may not be alleviated by using the topical anaesthesia only.
- A 5mm punch biopsy will be taken from the hypertrophic scar to be treated with laser therapy (intervention area, control area and one biopsy from normal skin). Please see study schedule (Appendix A).
- Specimens will be divded and stored in Formalin and -80^▫^C freezer as per SOP.
- The specimen will be labelled “Specimen for clinical trial” and with patient unique trial number. The histological analysis will be performed at the UoB Laboratory.
- The wound will be redressed with a simple non-adherent dressing. The punch biopsy will not be closed using sutures as healing will occur naturally in a few days.
- The samples will be preserved in University of Birmingham Biobanks used for research
- The tissue biopsies will be handled and used appropriately as per Human Tissue Act (2004)

**7.3.4 Subjective Scar Assessments**

Patients will be requested to complete subjective scar assessments at each time point: Vancouver Scar Scale (VSS), Patient and Observer Scar Assessment Scale (POSAS, version 2.0) and Brisbane Burn Scar Impact Profile (BBSIP, version 1.0). These questionnaires are part of the standard of care in evaluating burns patients.

**7.3.4.1 Vancouver Scar Scale**

A version of the VSS that was adapted from the modified version used by Nedelec et al is used in this study. This scale uses a numerical assessment of four skin characteristics including: Height (range, 0-4), Pliability (range, 0-4), Vascularity (range, 0-3), and Pigmentation (range, 0-3). The assessors choose a numerical value for each of these characteristics based on a comparison with normal skin.

**7.3.4.2 Patient and Observer Scar Assessment Scale**

POSAS questionnaire seeks to measure scar quality. POSAS, version 2.0 is a subjective scar scale that consists of two parts: a Patient Scale and an Observer Scale. Both scales contain six items that are scored numerically on a ten-step scale and together they make up the ‘Total Score’ of the Patient and Observer Scale.

The POSAS Patient scale assesses the scar in terms of pain, itching, scar colour, stiffness, thickness and irregularity, and overall opinion.

**7.3.4.3 Brisbane Burn Scar Impact Profile (BBSIP)**

The Brisbane Burn Scar Impact Profile (BBSIP, version 1.0) is a questionnaire that was developed to assess the health-related quality of life in people with burn scars. It consists of seven parts which measure the overall impact of burn scars; itch, pain and other sensations (and their impact on the subject); impact of the burn scars on work and daily activities; impact of the burn scars on relationships and social interactions; subjects perception of the impact of the burn scars on their appearance; their emotional reactions towards their scars; and physical symptoms caused by the burn scars.

**7.3.4.4 EQ-5D**

The EQ-5D is a questionnaire used for measuring generic health status. The EQ-5D questionnaire consists of 5 questions relating to different domains of quality of life (mobility, self-care, usual activities, pain/discomfort, anxiety/depression) for each of which there are 3 levels of response (no problems, some problems or severe problems). The NICE guidelines state that the EQ-5D questionnaire is the preferred measure of health related quality of life in adults. The study participant will complete the EQ-5D questionnaire.

**7.3.5 Objective Scar Assessments**

**7.3.5.1 DSM III Colormeter**

The DSM III Colormeter (Cortex Technology) is a small handheld device which combines two methods of quantifying colour: narrow-band spectrophotometry (melanin, erythema) and tristimulus reflectance colorimetry in a single measurement. It has a skin measuring area of .7mm in diameter. It consists of a handheld probe which utilises two high intensity white LED lights and accommodates the colour sensor, filters, and optics. . Measurements are done by placing the probe over the selected area on the scar. The probe is to be held perpendicular to the scar using minimal pressure to avoid blanching of the scar.

**7.3.5.2 Dermascan C USB**

The Dermascan C USB (Cortex Technology) is a high-frequency (20MHz) ultrasound scanner that enables the imaging of soft tissue at high resolution with a computer, and comes with software that allows automated skin thickness measurement. A medium focus transducer will be used with a 12mm wide viewing field and penetration depth of 15mm.

Before measurement, a thin layer of conducting ultrasound gel will be applied to the transducer and the transducer is to be held perpendicular to the scar sites to record echographic images for each site.

All measurements are to be performed with an ultrasound frequency set at 1580m/s. Thickness and density (as a measure of total intensity in percentage) measurements are then generated using the dedicated software (Advance Control 6 Analysis SW package, Cortex).

The thickness measured is defined as the distance between the echogenic stratum corneum and the inner surface of the dermis (in millimetres).

**7.3.5.3 Cutometer**

The cutometer (MPA 580, Courage and Khazaka) is an electronic instrument that assesses skin elasticity. The probe of the device is placed over the area of measurement, and then generates a negative pressure which draws the skin into a hollow aperture in the centre of the probe and then uses a laser to estimate the amount of skin displacement.

The probe with a 6-mm diameter hollow aperture was chosen for this study as previous studies have determined it to be the most efficient size to measure the visco-elasticity properties of the dermis^16-18^. For this study, mode 1 was chosen. This delivers three cycles of negative air pressure (500 mbar) for 2 seconds, followed by 2 seconds of no pressure. Results are expressed as the means of the three measurement cycles.

**7.3.5.4 Vectra H1 3D Camera**

The Vectra H1 handheld imaging system delivers high resolution 3D images for clinical use. Three-dimensional (3D) measurement systems are used to overcome the limitation of 2D photograph, mostly used for clinical documentation. They can be utilised to measure surface area of wounds, but additionally be able to measure the volume of scars much more quickly and easily compared to traditional methods such as moulding.

The Vectra 3D camera has shown to be superior to other systems with a higher resolution and the utilision of an adjustable light-beam pointer to aid positioning and does not require single use disposable targets.

##

## 7.4 Clinical and Laboratory Assessments

**7.4.1 Laboratory Assessment**

Once the blood and tissue samples have been taken by the clinician or researcher at either site, the samples will be sent to the University of Birmingham (UoB) Research Laboratories based at the Queen Elizabeth Hospital Birmingham. The UoB laboratory will initially process the bloods and run a rapid testing for COVID-19 as a safety measure before they proceed in processing bloods for research. UoB lab is currently processing confirmed COVID-ve bloods. If the blood results turn out COVID+ve, the blood samples will be discarded in accordance with the Trust policy in disposing biological samples.

The delegated staff at Morriston Hospital, Swansea, Wales will initially process the blood samples and store them at -80^0^C and then send them frozen at University of Birmingham (UoB) Laboratories based at the Queen Elizabeth Hospital Birmingham (QEHB). The processing and storage of blood samples will be discussed in detail in the laboratory manual. The tissue biopsies will also be processed and stored in formalin and -80^▫^C freezer. The frozen samples will be stored and sent in batch through a designated courier to the UoB laboratory based at QEHB.

All samples will be sent with a linked anonymous form with no patient identifiable data. The link to the patients will be held on a screening log in the research office based at the QEHB. Tissue samples will be sent to the University of Birmingham laboratory, where some will be processed and analysed and some will be transferred to the HBRC biobank for long-term storage.

Serial collection of peripheral blood and tissue biopsies will be performed as summarised in Figure 1. Each sample will be extracted according to the respective SOP.

The list given below illustrates the key laboratory tests that will be performed on participant samples. However, the list is not exhaustive and may be modified during the ongoing analysis of data throughout the study.

**7.4.1.1 Analysis of Tissue**

Tissue samples will be taken from normal skin, scarred skin treated with laser (treatment area) and scarred skin not treated with laser (control area) for analysis.

Fresh collected biopsies will be split in half and kept in formalin and -80^▫^C freezer, respectively.

Depending on this initial processing, biopsies will be transferred and stored in: Formalin – QEHB/HBRC (tissue bank) – HBRC will further process the formalin fixed tissue, and paraffin embed them. Frozen samples stored in -80^0^C freezer and Cell dissociation solution – processed on the same day for immunophenotyping purposes.

We aim to identify novel markers associated with scarring and tissue regenerative capabilities such as markers of cellular senescence and DNA damage, for example p16 and ɣH2Ax and other biomarkers including micro RNAs, TGF-beta, decorin, VAP-1, MMPs, PDGF, adiponectin, etc.

Tissue samples will also undergo staining and immunostaining as part of the histological analysis. H&E stain, Movat’s pentachrome stain, Herovici’s staining, amongst others, will be used to process epidermal and dermal height, inflammation, collagen orientation and thickness.

Immunostaining of tissue will be used to assess markers of inflammation and  regeneration, for example identification of types of collagen, smooth muscle actin, markers of angiogenesis or microvasculature (CD31), neutrophils (CD15), macrophages (CD68) and keratinocyte staining (CK 10, CK14) are to name a few.

The initial sample will provide a baseline histological profile of the scar tissue and subsequent samples will examine the collagen arrangement and fibrosis with and without laser therapy.

**7.4.1.2 Analysis of Blood**

Soluble markers will be sourced from blood which is processed to serum (6mls) and plasma (4.5 mls). For Morriston Hospital, an additional blood sample of 4.0 ml using EDTA tube will be collected for full blood count.  We aim to identify expressions and markers that influence scar formation, wound healing and variables of scar behaviour in response to CO_2_ laser therapy. This will include pro and anti-inflammatory cytokines, scar biomarkers and markers of cellular senescence, for example mmp.

Serum will be prepared by the centrifugation of blood collected into tubes containing z-serum clot activator. To measure plasma cfDNA, trisodium citrate anticoagulated blood will be 20 minute spin at 2,000 x g at 4 degrees and then collect the supernatant from the first spin and then spin at t 13,000 x g for 2 minutes at 4 degrees.

**7.4.2 Storage and disposal of samples**

This study will include the use of newly obtained blood (serum and plasma) and tissue samples provided by the participant. The samples will be collected by a trained research nurse or clinician. Once collected, the samples will be processed at the University of Birmingham by a member of the research team.

Aliquots of serum and plasma will be stored at -80°C in linked anonymised form in which the donor will not be identifiable to researchers. The samples will be stored in the University of Birmingham Laboratories based at the Queen Elizabeth Hospital.

Some of the skin biopsy samples will be stored in University of Birmingham biobanks. University Hospital Birmingham Samples will be used for various measurements as part of the study. Once the study has concluded, samples will be stored for future research in the biobanks.

All samples will be anonymised prior to transfer to the UoB Laboratory where they will be processed, stored and destroyed in accordance with the Human Tissue Act (2004) as described above. All samples will be kept until 10 years after analysis is completed.

**7.5 Schedule of Assessments and Data Collection**

**7.5.1 Baseline Data Collection (Recruitment/pre-assessment)**

Before any study related data or specimens are taken, the researcher will ensure that informed consent has been obtained and confirm that the trial subject meets the inclusion and non of the exclusion criteria. The study personnel will then collect and record the following information in the paper CRF booklet and eCRF (electronic case report form):

- Baseline patient information (age, sex, ethnicity, comorbidities, pre-injury medications)
- Burn/ trauma mechanism e.g. flame, scald etc.
- Anatomical location of scar to be treated with Laser therapy (wound map)
- Medical co-morbidities including Psychiatric or Behavioural Problems
- Subjective scar questionnaires - VSS, POSAS, BBSIP, QoL questionnaire (EQ-5D)
- Objective scar assessments – 3D camera, DSMII Colorimeter®, Dermascan® & Cutometer®.
- Clinical photograph of area of interest (treatment and control)
- Blood sample
- 2 Punch Biopsies (5mm) of scar tissue from identified sites that will be randomised for control and treatment area
- Punch Biopsy (5mm) of comparable normal skin.

**7.5.2 First laser intervention (Day 1)**

- Adverse events

**7.5.3 Post 1^st^ laser intervention (Day 22 +/- 3 days)**

- Clinical assessment and post laser care
- Clinical photographs
- Adverse events
- Punch Biopsy (5mm) only intervention area.
- Blood sample

**7.5.4 Second laser intervention (3 months +/- 30 days)**

The following clinical examinations and data will then be collected and recorded in the eCRF:

- Adverse events
- Blood sample
- Clinical photograph
- Subjective scar questionnaires – VSS, POSAS, BBSIP, EQ-5D
- Objective scar assessments – 3D camera, DSMII Colorimeter®, Dermascan® & Cutometer®

**7.5.5 Third laser intervention (6 months +/- 30 days)**

The following clinical examinations and data will then be collected and recorded in the eCRF:

- Adverse events
- Punch Biopsy (5mm) of intervention area (Pre 3^rd^ intervention) only. (No control or normal skin biopsies.)
- Blood sample
- Clinical photograph
- Subjective scar questionnaires – VSS, POSAS, BBSIP, EQ-5D
- Objective scar assessments – 3D camera, DSMII Colorimeter®, Dermascan® & Cutometer®

**7.5.6 Follow-up Visit (6 months post 3^rd^ ^t^ Laser Treatment +/- 30 days)**

The following clinical examinations and data will then be collected and recorded in the eCRF:

- Adverse events
- Punch biopsy 5mm of **both** the intervention and control areas.
- Blood sample
- Clinical photograph
- Subjective scar questionnaires - VSS, POSAS, BBSIP, EQ-5D
- Objective scar assessments – 3D camera, DSMII Colorimeter®, Dermascan® & Cutometer®

# DATA MANAGEMENT & STATISTICS

## Determination of Sample Size

**8.1.1 Laser Treatment and Standard of Care**

Following recommendations for pilot studies, 30 patients or more are typically required to gain estimates of the parameters needed for sample size estimation. No formal sample size calculation has been performed as a result. We have allowed for a 20% drop-out and possible loss to follow-up. We therefore aim to recruit 60 participants in total. This will also allow the recruitment and retention rates to be estimated with 95% confidence interval maximum widths of 20% and 25% respectively.

## Statistical Analysis

The primary comparison groups will be the scar sections randomised to (control group) versus those randomised to treatment with laser therapy (experimental group). Outcome data will be collected at pre-laser, 3 weeks post the first and third treatments and 12 month

s post third treatment.

All analysis will be based on the intention to treat principle, i.e. all scars will be analysed in the treatment group to which they were randomised irrespective of compliance with the allocated treatment or other protocol deviation. The data analysis for this pilot trial will be descriptive and mainly focus on confidence interval estimation, with no formal hypothesis testing performed.

Dichotomous feasibility measures, like the recruitment and retention rates, including completeness of data will be reported as numbers and percentages. These values will be summarised across participants or treatment groups as appropriate.

Given this study is a pilot, the data will help inform the selection of the most appropriate primary outcome measure for a larger RCT and provide data to estimate the sample size required for the main RCT.

Analysis methods will be chosen according to the data type; 1. Continuous endpoints (e.g. VSS total score): These data will be summarised using means and standard deviations, with differences in means with 95% confidence intervals reported. Longitudinal plots of the data over time will also be constructed for visual presentation of the data. 2. Categorical (dichotomous) endpoints (e.g. rates of improvement in scar domains): The number and percentages of participants/scars experiencing the event will be summarised across and between groups.

As this is a randomised study, there are no priori subgroup analyses specified.

Every attempt will be made to collect full follow-up data on all study participants with the anticipation that missing data will be minimal. The assessment of missing data is an outcome measure of this study.

If a suitable primary outcome is identified during the pilot trial, the level of missing data will form one component of the assessment of feasibility for a future trial. As this is a pilot trial, no formal sensitivity analysis will be conducted.

The primary analysis for the trial will occur once all participants have completed the 12 month assessment and corresponding outcome data has been entered onto the trial database, validated as being ready for analysis, and the database locked. This analysis will include data items up to and including the 12 month assessment.

For the secondary outcome data about the biomarkers of the scar response to laser therapy the various cellular and molecular variables will be examined for relationships to scarring using logistic regression. Further longitudinal analysis to identify potential drivers of a positive response will be done using linear mixed-effects modelling.

### 8.3 Psychometric Data Analysis

**8.3.1** We would like to recruit sample size of 100 to 150 to ensure stability of person and item calibrations in the psychometric data analysis. (Linacre, 1994) To enable item calibrations on the PROMs to within ½ logit requires 100 to 150 participants for confidence intervals of between 95% and 99% this will help us ensure that we have sufficient power to test out the psychometric validity of the PROMs being used in the SMOOTH study.

Data will be analysed using Classical Test Theory (CTT) and Rasch Analysis to capture change over time and comparisons using known groups validity. A small cohort of approximately 60 people will be asked to complete the measure 2 weeks apart to evaluate for test-retest reliability.

Rasch Analysis to identify psychometric validity of measures:

**8.3.2 The Rasch Model**

The Rasch model can be utilised to examine the psychometric properties of the BBSIP that cannot always be identified using traditional analysis such as CTT. The Rasch model discloses anomalies in the data and the extent to which individual items in the BBSIP capture a unidimensional construct and therefore the extent to which summation of items from the PROMS are legitimate (19).

**8.3.2.1 Rasch Analysis**

Rasch analysis is an iterative process that will identify anomalies in the data and the extent to which the PROMs data conforms to the Rasch model and therefore the extent to which the instruments are unidimensional. Fit will be established using a variety of indicators and fit statistics. The Rasch Unidimensional Measurement Model software (RUMM2030) will be used to analyse the PROMs. (RUMMLAB, 2018, Andrich, 1988)

**8.3.2.2 Scoring Categories**

Rasch analysis can examine the scoring categories of the instruments and establish the extent to which scoring categories capture increasing/decreasing levels of the underlying construct. Where affirmation of scoring categories by respondents follows a logical sequence then Rasch Andrich thresholds appear ordered. Rasch Andrich thresholds are the points between adjoining categories where the probability of affirming either category is 50/50, when responders’ perceived level of health is equidistantly captured by adjoining categories. Where there is agreement with this expected response hierarchy, thresholds appear ordered, disordered thresholds are observable as a lack of consistency. Disordered thresholds can suggest poorly defined or redundant scoring categories and therefore conceptual distinctions between categories maybe imprecise. Responders then find it difficult to assign a category to their perceived health status.

**8.3.2.3 Targeting**

Targeting will be established by examining the extent to which distributions of participants perceived health status and levels of health identified by the PROMs are analogous.

**8.3.2.4 Person Separation Index**

The person separation index (PSI) is conceptually equivalent to Cronbach’s Alpha. It identifies the extent to which the instrument is able to discriminate between groups with different health states and the precision of the estimate for each person.

**8.3.2.5 Tests of fit**

Individual tests of fit for each person and item will reflect the difference between responders observed and expected responses if data fits the Rasch model. RUMM2030 automatically clusters responders into equivalent size groups (class intervals) according to their overall level of perceived health. A number of statistics utilise these class intervals including χ^2^ statistics and residual values (±2.5). Residuals are summations of individual item or person deviations from expected fit to the Rasch model, standardised as a z-score. Residual scores between ± 2.5 indicate adequate fit to the Rasch model. A significant chi-square suggests data do not fit the Rasch model.

**8.3.2.6 Differential Item Functioning**

Differential item functioning (DIF) is a form of item bias that can affect fit to the model. DIF manifests itself when responses to individual items by sample sub-groups (e.g. gender or age group) are inconsistent with their overall perceived level of health. DIF will be identified using ANOVA and statistically significant probability (*p*<.05, or a Bonferroni corrected level). DIF for gender, age group and additional identified clinical group categories e.g. type of burn injury will be examined.

**8.3.2.7 Item Independence**

Item independence is an underlying principle of the Rasch Model. Response dependency occurs when a person’s response to one item determines the response to another item and therefore responses are not independent of each other. Residual correlation metrics (<0.2 above the average residual correlation) will identify if response dependency is an issue.

Once the ‘Rasch factor’ is extracted, leftover residuals should not contain any patterns in the data. A principal components analysis (PCA) of the residuals will detect if multi-dimensionality is an issue.

**8.3.2.8 Logit transformation**

If data from the individual measures fits the Rasch model, patient and item parameter estimates are then positioned on the same log-odds units (logits) scale, although as independent parameters. This allows for a linear transformation of the raw scores to be utilised. Therefore, estimates of a patient’s perceived perceptions relating to their scars or impact of scarring on quality of life can be derived from the PROMs with confidence.

**8.3.2.9 Modify Instrument**

Based on the results of the psychometric validation of the PROMS, modifications may be required to ensure that the PROMs capture the best quality data.

## 8.4 Data Monitoring & Interim Analysis

Data analysis will be performed on a 3 monthly basis to ensure that high quality, relevant data is being generated. If some blood sampling is felt of little benefit or shows little correlation this may be discontinued. This will ensure that patients are not undergoing unnecessary blood sampling. The decision to remove a specific test will be made following collaboration with all Investigators

## 8.5 Direct Access to Source Data/Documentation

Patient’s confidentiality will be maintained at all times. Following receipt of patients consent, each patient will be allocated with a trial specific number. Patents will not be referred to during the trial by name or hospital unit number. The non-identifiable trial specific number will be used at all times to ensure patient confidentiality. A record of trial numbers and patient details will be held at the Queen Elizabeth Hospital Research Office and will only be known to the principal investigators and research personnel. The patient data will not be supplied to the sponsor. However, during the study, representatives from the sponsor, and representatives of regulatory agencies such as the ethics committee, will be allowed to review patient medical records that relate to the study. The records will identify patients only by the assigned trial specific number.

## 8.6 Data Handling and Record Keeping

The Principal Investigator will be responsible for the secure storage of all trial related documentation. All documentation including copies of protocols, patient’s information leaflets, GP letters, consent forms, and CRFs will be held securely in accordance with current ICH GCP guidelines for a minimum of fifteen years. The records will be available for review by governing bodies upon request following notice.

## 8.7 Quality Control and Quality Assurance

To ensure the production of high quality data, all research staff will be familiar and adequately trained according to the Good Clinical Practice guidelines. Data will be managed by a specific research team familiar with the research trial and the research protocol.

# ETHICS

This research trial will be conducted according to the Declaration of Helsinki (2008). The protocol and all supporting information including the patient information leaflets and consent forms will be submitted to the ethics committee and local research and development department for review and prior to any commencement of the trial. The trial will only begin once required approvals have been granted.

# FINANCE AND INSURANCE

The research trial is a joint collaboration between the University Hospital Birmingham and the University of Birmingham. Funding for the delivery of the study is supported by the Chancellor using LIBOR funds via a grant obtained by The ScarFree Foundation.

NHS bodies are legally liable for the negligent acts and omissions of their employees. If participants are harmed whilst taking part in a clinical trial as a result of negligence on the part of a member of the study team this liability cover would apply.

Non-negligent harm is not covered by the NHS indemnity scheme. The University Hospitals Birmingham NHS Foundation Trust, therefore, cannot agree in advance to pay compensation in these circumstances.

# PUBLICATION POLICY

The findings from the research trial will be published at various stages of the trial in relevant medical journals and presented at medical conferences.

# ARCHIVE PLAN

All data and trial related information collected throughout the duration of the study will be stored for 15 years after the completion of the study. The PI or designee must maintain adequate and accurate records to enable the conduct of the Trial to be fully documented and the Trial data to be subsequently verified. After trial closure the PI will maintain all source documents and trial related documents. All trial documents will be archived in accordance with the UHBFT Archiving Procedures.

**References:**

1. Gangemi EN, Gregori D, Berchialla P, Zingarelli E, Cairo M, Bollero D, et al. Epidemiology and risk factors for pathologic scarring after burn wounds. Archives of facial plastic surgery. 2008;10(2):93-102.

2. Manstein D, Herron GS, Sink RK, Tanner H, Anderson RR. Fractional photothermolysis: a new concept for cutaneous remodeling using microscopic patterns of thermal injury. Lasers in surgery and medicine. 2004;34(5):426-38.

3. Zuccaro J, Ziolkowski N, Fish J. A Systematic Review of the Effectiveness of Laser Therapy for Hypertrophic Burn Scars. Clinics in plastic surgery. 2017;44(4):767-79.

4. Gauglitz GG, Korting HC, Pavicic T, Ruzicka T, Jeschke MG. Hypertrophic scarring and keloids: pathomechanisms and current and emerging treatment strategies. Molecular medicine (Cambridge, Mass). 2011;17(1-2):113-25.

5. Slemp AE, Kirschner RE. Keloids and scars: a review of keloids and scars, their pathogenesis, risk factors, and management. Current opinion in pediatrics. 2006;18(4):396-402.

6. Shaw AC, Joshi S, Greenwood H, Panda A, Lord JM. Aging of the innate immune system. Current opinion in immunology. 2010;22(4):507-13.

7. Gunin AG, Kornilova NK, Petrov VV, Vasil'eva OV. [Age-related changes in the number and proliferation of fibroblasts in the human skin]. Advances in gerontology=Uspekhi gerontologii. 2011;24(1):43-7.

8. Coppe JP, Desprez PY, Krtolica A, Campisi J. The senescence-associated secretory phenotype: the dark side of tumor suppression. Annual review of pathology. 2010;5:99-118.

9. Demaria M, Ohtani N, Youssef SA, Rodier F, Toussaint W, Mitchell JR, et al. An essential role for senescent cells in optimal wound healing through secretion of PDGF-AA. Developmental cell. 2014;31(6):722-33.

10. Draaijers LJ, Tempelman FR, Botman YA, Tuinebreijer WE, Middelkoop E, Kreis RW, et al. The patient and observer scar assessment scale: a reliable and feasible tool for scar evaluation. Plastic and reconstructive surgery. 2004;113(7):1960-5; discussion 6-7.

11. Tyack Z, Kimble R, McPhail S, Plaza A, Simons M. Psychometric properties of the Brisbane Burn Scar Impact Profile in adults with burn scars. PloS one. 2017;12(9):e0184452.

12. Griffiths C, Guest E, White P, Gaskin E, Rumsey N, Pleat J, et al. A Systematic Review of Patient-Reported Outcome Measures Used in Adult Burn Research. Journal of burn care & research : official publication of the American Burn Association. 2017;38(2):e521-e45.

13. Jones LL, Calvert M, Moiemen N, Deeks JJ, Bishop J, Kinghorn P, et al. Outcomes important to burns patients during scar management and how they compare to the concepts captured in burn-specific patient reported outcome measures. Burns : journal of the International Society for Burn Injuries. 2017;43(8):1682-92.

14. Andrews N, Jones LL, Moiemen N, Calvert M, Kinghorn P, Litchfield I, et al. Below the surface: Parents' views on the factors that influence treatment adherence in paediatric burn scar management - A qualitative study. Burns : journal of the International Society for Burn Injuries. 2017. Oct 12. pii: S0305-4179(17)30482-5. doi: 10.1016/j.burns.2017.09.003. [Epub ahead of print]

15. Lee KC, Dretzke J, Grover L, Logan A, Moiemen N. A systematic review of objective burn scar measurements. Burns & trauma. 2016;4:14.

16. Brusselaers N, Pirayesh A, Hoeksema H, Verbelen J, Blot S, Monstrey S. Burn scar assessment: A systematic review of objective scar assessment tools. Burns : journal of the International Society for Burn Injuries. 2010;36(8):1157-64.

17. Rothman ML, Beltran P, Cappelleri JC, Lipscomb J, Teschendorf B. Patient-reported outcomes: conceptual issues. Value in health : the journal of the International Society for Pharmacoeconomics and Outcomes Research. 2007;10 Suppl 2:S66-75.

18. Patrick DL, Burke LB, Powers JH, Scott JA, Rock EP, Dawisha S, et al. Patient-reported outcomes to support medical product labeling claims: FDA perspective. Value in health : the journal of the International Society for Pharmacoeconomics and Outcomes Research. 2007;10 Suppl 2:S125-37.

19. Pallant JF, Tennant A. An introduction to the Rasch measurement model: an example using the Hospital Anxiety and Depression Scale (HADS). The British journal of clinical psychology. 2007;46(Pt 1):1-18.

20. Lancaster GA, Dodd S, Williamson PR. Design and analysis of pilot studies: recommendations for good practice. Journal of evaluation in clinical practice. 2004;10(2):307-12.

21. Browne RH. On the use of a pilot sample for sample size determination. Statistics in medicine. 1995;14(17):1933-40.

| Appendix A. Study Schedule | **Recruitment^10^**  **/ Pre-Assessment^11^** | **1^st^ treatment^12^**  **(Day 1)** | **3 weeks^13^ Post 1^st^ treatment**  **(Day 22+/-3days)** | **2^nd^ Treatment^14^**  **(Month 3 +/-30 days)** | **3^rd^ Treatment^15^**  **(Month 6 +/-30 days)** | **6 months post 3rd treatment**  **(+/-30 days)** |
| --- | --- | --- | --- | --- | --- | --- |
|  |  |  |  |  |  |  |
|  |  |  |  |  |  |  |
| Informed Consent | **x** |  |  |  |  |  |
| Inclusion and Exclusion Criteria | **x** |  |  |  |  |  |
| Medical History ^1^ | **x** |  |  |  |  |  |
| Adverse Events |  | **x** | **x** | **x** | **x** | **x** |
| Concomitant Medications | **x** | **x** | **x** | **x** | **x** | **x** |
| Registration & Randomisation | **x** |  |  |  |  |  |
| Screen for pregnancy ^2^ | **x** | **x** |  | **x** | **x** |  |
| **Burn Assessment** |  |  |  |  |  |  |
| Record Burn injury details ^3^ | **x** |  |  |  |  |  |
| Record Treatment Site ^4^ | **x** | **x** | **x** | **x** | **x** | **x** |
| Clinical photography ^5^ | **x** |  | **x** | **x** | **x** | **x** |
| **Treatment ^6^** |  |  |  |  |  |  |
| Laser Therapy ^7^ |  | **x** |  | **x** | **x** |  |
| **Scar Assessments** |  |  |  |  |  |  |
| 3D Vectra Camera photography | **x** |  |  | **x** | **x** | **x** |
| Dermascan® ultrasound | **x** |  |  | **x** | **x** | **x** |
| Cutometer® | **x** |  |  | **x** | **x** | **x** |
| DSM II colormeter® | **x** |  |  | **x** | **x** | **x** |
| Modified Vancouver Scar Scale (mVSS) | **x** |  |  | **x** | **x** | **x** |
| Patient and Observer Scar Assessment Scale (POSAS) | **x** |  |  | **x** | **x** | **x** |
| Brisbane Bars Scar Impact Profile Score (BBSIP) | **x** |  |  | **x** | **x** | **x** |
| Quality of Life Questionnaire (EQ-5D) | **x** |  |  | **x** | **x** | **x** |
| **Laboratory Tests** |  |  |  |  |  |  |
| Blood sample ^8^ | **x** |  | **x** | **x** | **x** | **x** |
| Normal skin biopsy ^9^ (Baseline) | **x** |  |  |  |  |  |
| Scar Non-laser treated biopsy ^9^  (Control) | **x** |  |  |  |  | **x** |
| Scar treated with laser biopsy ^9^ (Randomised) | **x** |  | **x** |  | **x^16^** | **x** |

1. Medical History - include patient demographics e.g. age, sex, ethnicity, medical co-morbidities including Psychiatric or Behavioural Problems and History of alcohol or substance misuse
2. Screen for pregnancy - pregnancy test not routinely performed unless suspected
3. Record Burn injury details - include % Total Body Surface Area (TBSA) Burned or surface area traumatised (calculated by Lund & Browder Chart), Burn/ trauma mechanism e.g. flame, scald etc,
4. Record Treatment site - Anatomical location of scar to be treated with Laser therapy (wound map)
5. Clinical Photography - of area of interest - randomised/treatment and control areas
6. Treatement - 3 months intervals post recruitment (+/-30 days)
7. Laser Therapy - use a Lumenis® UltraPulse® CO2 laser device with the SCAARFX^TM^: Energy 110-150 mJ; Density 3%; Shape: setting 2; Size: setting 10; Pulses 1; and DeepFX^TM:^ Energy 17.5-20.0 mJ; Density 5%; Shape: setting 2; Size: setting 10;; Pulses 1; Repeat rate 0.5 - 1 seconds; Frequency 250 - 300Hz; No active cooling will be used during the treatment.
8. Bloods –maximum 15mls: at QEHB = x1 Blue top 4.5ml plasma + x1 Red top 6ml serum ; at Morriston Hospital = x1 blue top 4.5 ml plasma, x1 4.0 ml EDTA (purple top) tube; x1 red top 6 ml serum; additional 1 ml blood green top lithium heparin for COVID-19 testing.
9. Biopsy - 5mm punch biopsy
10. Recruitment - Day 0 of study
11. Pre Laser Assessment - Baseline assessment prior to first laser treatment
12. 1^st^ Treatment - Day 1 - Day of first laser intervention
13. 3 weeks post treatment Assessement - +/-3days allowance
14. 2^nd^ Treatment – Month 3 - Laser treatement at 3 months +/- 30days after the 1^st^ treatment
15. 3^rd^ Treatment - Month 6 - Laser treatement at 6 months +/- 30days after the 1^st^ treatment
16. Third Treatment Visit - 5mm punch biopsy to be performed, i.e., before the third laser therapy
