# Supplementary material for: SMOOTH protocol: A pilot randomised prospective intra-patient single-blinded observational study for examining the mechanistic basis of ablative fractional carbon dioxide laser therapy in treating hypertrophic scarring
Source: PLoS One. 2023 Sep 8;18(9):e0285230. doi: 10.1371/journal.pone.0285230 (PMC10490849; doi:10.1371/journal.pone.0285230)
Supplement: S1 Table — (DOCX) [file pone.0285230.s005.docx]

**S1 Table** Trial registration dataset

| Data category | Information |
| --- | --- |
| Primary registry and trial identifying number | ClinicalTrials.gov  NCT04736251 |
| Date of registration in primary registry | 2020-10-27 |
| Secondary identifying numbers | RRK6716 |
| Source(s) of monetary or material support |  |
| Primary sponsor(s) | University Hospital Birmingham Foundation Trust |
| Contact for public queries | Ms Minnie Ventura |
| Public title | Mechanistic Basis of Ablative Carbon Dioxide Laser in Treating Hypertrophic Scars, SMOOTH |
| Scientific title | A prospective Intra-patient **S**ingle-blinded randomised trial to examine the **M**echanistic basis of fracti**O**nal ablative carb**O**n dioxide laser therapy in treating adult burns and/or trauma patients with hypertrophic scarring |
| Countries of recruitment | United Kingdom |
| Health condition(s) of problem(s) studied | Scar  Burn scar |
| Intervention(s) |  |
| Key inclusion and exclusion criteria | Inclusion Criteria:   - Adult patients aged = 16years - Patient with hypertrophic scarring as a result of deep dermal or full thickness burns/trauma. - Trauma or burn sustained more than 12 months prior to recruitment. - Treatment area to be =25cm^2^ confluent scarring with a comparable control scar on limb or trunk.   General Exclusion Criteria:   - Patients under 16 years of age. - Previous laser therapy treatment to the study site. - The use of recent (within 6 months) or concurrent invasive scar treatments, including intra-lesional pharmaceuticals, micro needling or other laser modalities (e.g. pulse-dye.). - Known allergy or contraindication to eutectic mixture of local anaesthesia (ELMA) cream (lidocaine 2.5% and prilocaine 2.5%), components of moisturising cream (benzalkonium chloride 0.1%; chlorhexidine dihydrochloride 0.1%; liquid paraffin 2.5%; isopropyl myristate 2.5%) or ointment (white soft paraffin liquid paraffin %w/w 50 50.). - Patients with Fitzpatrick skin type of 5-6 due to nature of the skin.   Laser Treatment Exclusion Criteria   - The presence of acute infection at the proposed treatment site. - Pregnancy or lactation. - Patients with poorly controlled Diabetes mellitus HbA1c >9% or 75mmol/mol within last 3 months). - Patients experiencing acute exacerbation of chronic skin diseases e.g. psoriasis or eczema. - Immunosuppression (HIV, drugs with immunosuppressive effect). - Use of Roaccutane at any time within the last 6 months. - Autoimmune disorders in active stage (for example: 1. Localised; Type 1 diabetes mellitus, Addison's, Grave's and Crohn's Disease, 2. Systemic; Rheumatoid arthritis, multiple sclerosis, lupus and scleroderma). - Known history of keloid scarring. |
| Study type | Observational |
| Date of first enrolment | December 9, 2019 |
| Target sample size | 60 |
| Recruitment status | Recruiting |
| Primary outcome(s) | Detect the number of senescent cells and the sub-population of fibroblasts after the last CO_2_ laser therapy [Time Frame: 12 months].   - Measuring the proportion of senescent cells and the proportion and sub-population of fibroblasts following treatment to determine positive effect of CO_2_ laser therapy through histological assessment: To assess changes in the proportion of senescent cells (marker: p16) and types of fibroblasts (αSMA and CD90/Thy1) from baseline at 3 weeks, 6 months and 12 months after 1^st^ laser treatment. |
| Key secondary outcomes | - Quantify scar thickness [Time Frame: 12 months] - Quantify scar colour [Time Frame: 12 months] - Quantify scar elasticity [Time Frame: 12 months] - Quantify scar volume [Time Frame: 12 months] - Vancouver Scar Scale [Time Frame: 12 months] - Novel markers associated with scar formation, wound healing and variables of scar behaviours in response to CO2 laser therapy [Time Frame: 12 months] - Patient and Observer Scar Assessment Scale (POSAS) [Time Frame: 12 months] - Brisbane Burn Scar Impact Profile (BBSIP) [Time Frame: 12 months] - Health status [Time Frame: 12 months] |
